# Intrapericardial fibrinolysis and video-assisted thoracoscopic surgery in managing severe pediatric *Streptococcus pneumoniae* purulent complications

**DOI:** 10.36416/1806-3756/e20240127

**Published:** 2024-07-29

**Authors:** Jinghui Jiang, Qianqian Du, Jun Liang, Kaihu Yao

**Affiliations:** 1. Liaocheng People’s Hospital, Shandong 252000, China.; 2. Beijing Pediatric Research Institute, Beijing Children’s Hospital, Capital Medical University, National Center for Children’s Health, Beijing, 100045, China.

## DEAR EDITOR:

Confirmed *Streptococcus pneumoniae* infections in sterile body cavities, such as pediatric purulent pericarditis and typical pleural empyema, were relatively rare in the post-antibiotic and post-vaccine era.^1^ ^-^ ^3^ Inappropriate treatment selection could leave constrictive pericarditis or constrictive pleurisy, which have a considerable bearing on the long-term prognosis of affected children.^4^ We herein reported two cases featuring definitive etiological evidence and characteristic clinical presentations, with distinct therapeutic approaches employed to address the purulent effusions; both yielded favorable outcomes.

The first case (a 2-year-old female) was admitted under the primary diagnosis of pericardial effusion and sepsis. CT imaging disclosed the presence of pneumonia, bilateral pleural effusions, and a substantial pericardial effusion (Figure A1). *S. pneumoniae* was isolated from the patient’s blood and pericardial effusion cultures, which was subsequently identified as serotype 6A (ST9789) at Beijing Children’s Hospital. Unfortunately, cardiac ultrasound showed cellulose-like material in the pericardium (Figure A2). The patient underwent a series of eight intrapericardial fibrinolysis procedures. Urokinase was administered daily at a dose of 8 mL containing 40,000 units, which was directly infused into the pericardial cavity. A cumulative volume of 510 mL of pericardial fluid was drained throughout treatment. Echocardiography demonstrated a marked reduction in pericardial effusion volume, with only a negligible amount remaining (Figure A3).

**Figure 1:**
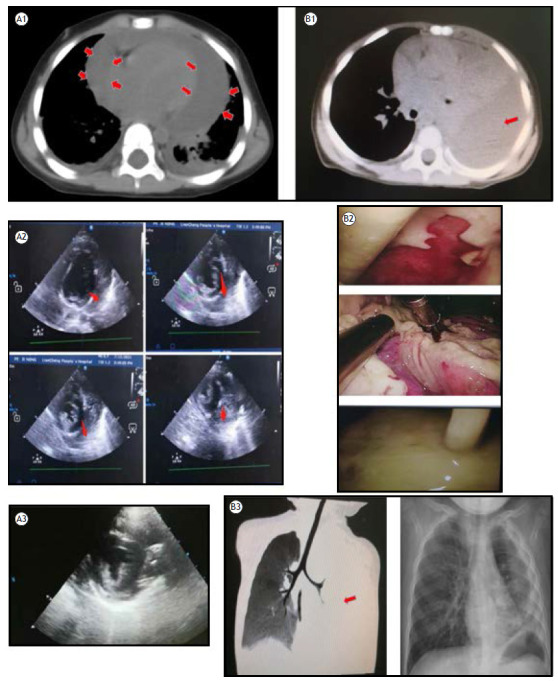
In A1, a chest CT scan showed the presence of pneumonia, bilateral pleural effusions, and substantial pericardial effusion (red arrow). In A2, cardiac ultrasound showed cellulose-like material in the pericardium. In A3, an echocardiography image showing the negligible amount of pericardial effusion remaining. In B1, a CT scan showing consolidation in the left lung, atelectasis areas, and pleural effusion (red arrow); In B2, the pleural cavity’s fibrous purulent material and pus could be cleared under direct vision. In B3, the left picture was a CT scan at admission, and the right picture was a CT scan conducted four months later confirming the child’s complete recovery.

On admission with severe dyspnea, the second child (1 year old) had a white blood cell count of 35.9 × 10^9^/L, accompanied by a C-reactive protein level exceeding 200 mg/L. CT revealed consolidation in the left lung, atelectasis areas, and pleural effusion (Figures B1 and B3, left). Microbiological analyses confirmed the presence of *S. pneumoniae* in blood, sputum, and pleural fluid cultures. Despite receiving intravenous antibiotics (azithromycin and cephalosporin) for a cumulative duration of 10 days, the patient’s clinical symptoms and blood biomarkers failed to demonstrate adequate improvement. Ultrasound examination of the chest revealed a substantial left-sided pleural effusion characterized by a profuse, grid-like septation pattern. The drainage volume was small and contained flocs or cord-like material, which prevented continuous drainage of the effusion. VATS was performed on the left side on the fifth day of admission, and the abscess and effusion in the left pleural cavity were carefully removed. The pleural cavity’s fibrous purulent material and pus could be cleared under direct vision (Figure B2). VATS removed the thick fiber separation that hindered the sufficient drainage of pleural effusion by the thoracic duct. A CT scan conducted four months later confirmed the child’s complete recovery to normalcy, with no discernible sequelae (Figure B3, right).

The British Thoracic Society and the American Pediatric Surgical Association guidelines^5^ ^,^ ^6^ suggested a trial of fibrinolytic therapy as the initial treatment of choice, followed by VATS for those failing pleural drainage with a fibrinolytic. This recommendation was founded on a lack of empirical data, yet we acknowledge the appropriateness of either therapeutic strategy in our two cases. Institutional expertise, economic constraints, and patient preferences may sway the ultimate decision.

Fibrin deposition, triggered by *S. pneumoniae*, seems pivotal in progressing towards constrictive pericarditis/pleuritis and perpetuating the disease state. The effectiveness of fibrinolytic agents (streptokinase and urokinase) in degrading extravascular fibrin has been well-established. Upon administering intrapericardial urokinase in the first case, the drainage volume of the patient escalated from 15 mL to 135 mL on the initial post-treatment day. Given the inherent risks of morbidity associated with pericardiectomy, intrapericardial fibrinolysis during pericardiocentesis emerges as a potentially less invasive alternative for averting the persistence of purulent pericarditis and the development of constrictive pericarditis.^7^ Minimally invasive treatment modalities are crucial in pediatric populations, as they typically offer superior safety profiles and enhanced feasibility. Urokinase has undergone a placebo-controlled investigation in pediatric populations; 40,000 units in 40 mL 0.9% saline in children aged one year and older was recommended by the British Thoracic Society^5^ and the Pediatric Infectious Diseases Society.^8^ It appeared to be safe and effective in preventing the development of pericardial constriction.

Surgical intervention may be employed as a first-line treatment modality for patients presenting with loculated pleural effusions/empyema or as a secondary therapeutic option for those who fail to respond satisfactorily to fibrinolytic therapy. Early engagement of a surgeon in the clinical decision-making process was instrumental in ensuring that any necessary surgical intervention could be promptly executed should the circumstances dictate. When surgical decortication was required, maximizing the benefits while minimizing physiological impact was more feasible through early intervention, swift progression to advanced treatment modalities, and management at a facility proficient in performing VATS.^9^ VATS may be indicated in loculated empyema.

Upon the child’s admission, we promptly solicited the expert opinion of a pediatric surgeon, who advised that surgical intervention should be pursued if the conservative treatment proved ineffective. Relative to the first case, this second patient received long-term intravenous antibiotics treatment and exhibited a more protracted clinical course and more pronounced symptomatology. According to the Pediatric Infectious Diseases Society recommendations,^8^ VATS should be selected directly if closed drainage is ineffective. The child recovered and was discharged 14 days after the operation and recovered well during the follow-up for the next 4 months. The second case aligned with the understanding that VATS was suitable for localized empyema.^10^ There was no recurrence and no constrictive pleurisy, indicating that timely treatment can help shorten the course of the patient’s illness and reduce costs.
